# The complex phenomenon of dysrational antibiotics prescribing decisions in German primary healthcare: a qualitative interview study using dual process theory

**DOI:** 10.1186/s13756-019-0664-6

**Published:** 2020-01-06

**Authors:** Regina Poss-Doering, Martina Kamradt, Anna Stuermlinger, Katharina Glassen, Petra Kaufmann-Kolle, Edith Andres, Michel Wensing

**Affiliations:** 1 Department of General Practice and Health Services Research, University Hospital Heidelberg, Im Neuenheimer Feld 130.3, 69120 Heidelberg, Germany; 2 aQua Institut, Maschmuehlenweg 8-10, 37073 Goettingen, Germany

**Keywords:** Antimicrobial resistance, Rational antibiotics use, Non-complicated infections, Upskilling, Dual process theory

## Abstract

**Background:**

Antibiotic prescription rates in primary care in Germany are moderate, but still considered too high. The ARena study (Sustainable reduction of antibiotic-induced antimicrobial resistance) was initiated to foster awareness and understanding of the growing challenge and promotes rational antibiotics use for acute, non-complicated and self-limiting infections.

**Methods:**

The present study was performed as part of the process evaluation of the ARena study. Interviews were conducted with a purposive sample of physicians participating in the ARena study to identify factors relevant to primary care physicians’ decision-making when prescribing antibiotics for acute non-complicated infections. Generated data were audio-recorded. Pseudonymized verbatim transcripts were coded using a pre-defined framework. The Dual Process Theory was applied to provide understanding of individual health professional factors that induce dysrational prescribing decisions.

**Results:**

Based on medical as well as non-medical considerations, physicians developed habits in decision making on antibiotics prescribing. They acknowledged inadequate antibiotics prescribing for acute, non-complicated infections in situations involving uncertainty regarding diagnosis, prognosis, continuity of care, patient expectations and when not knowing the patient. Educative efforts empowered physicians to override habitual prescribing. A theory-driven model provides transparency as to how dysrational prescribing decisions occur and suggests remedy by providing new experiences and new recognizable patterns through educative efforts.

**Conclusions:**

Educational interventions may only change prescribing behaviours if they result in active rational rather than routine-based decision-making on antibiotics prescribing.

**Trial registration:**

ISRCTN, ISRCTN58150046.

## Background

Antimicrobial resistance (AMR) is a naturally occurring process, driven by diverse factors. Research suggests that it is accelerated by misuse and overuse of antibiotics in people and livestock [1]. To meet concomitant challenges for modern healthcare, comprehensive global, European and German action plans have been developed [2–4]. A European comparison shows that the usage of antibiotics is moderate in Germany [5] and declining [6]. This may be related to scientific research on rational use of antibiotics and the implementation of evidence-based practice guidelines in the previous decade [7]. Nevertheless, the volume of inappropriate, non-indicated antibiotics prescribed in German ambulatory care remains too high, particularly the consumption of broad-spectrum antibiotics such as cephalosporins and fluoroquinolones [5, 8, 9], leaving substantial room for improvement [10].

Addressing antibiotics overprescribing requires profound understanding of physicians’ decision-making processes leading to inappropriate prescribing and a gap between knowledge and practice. The randomized trial ARena (Sustainable reduction of antibiotic-induced antimicrobial resistance, 2017–2020) aims to promote a rational and appropriate use of antibiotics for acute, non-complicated infections in primary care in Germany [10]. As ARena is not completed yet, the assessment of the effectiveness of this intervention is still pending.

However, the present qualitative study was part of the process evaluation conducted during the ARena trial. While ARena is ongoing, this qualitative study aimed to identify factors associated with primary care physicians’ decision-making processes. It looked beyond descriptive approaches and focused on theory-based factors that might induce non-indicated, inappropriate antibiotics prescriptions for acute non-complicated self-limiting infections.

The Dual Process Theory posits that generally two types of processing approaches can be activated in human decision making [11, 12] and in diagnostic reasoning [11, 13, 14]. Type 1 processing is regarded as fast, heuristic and intuitive, Type 2 processing as slow, systematic, analytical and logical. Type 1 can be triggered by individual perceptions, images, emotions and domain-specific context. It may be dysrational, leading to a mismatch between an automated pattern recognition and a cognitively controlled, rule-based decision-making process. Type 2 can be triggered by non-familiar, unrecognized presentation of subject matters and lead to a conscious reflection based on internalized knowledge, applicable guidelines and logic. Experts preferentially engage in Type 1 processing while novices tend to calibrate available options in Type 2 processing mode [13]. The aim of our study was to delineate a theory-driven model that identifies and explains deviations from rational and appropriate antibiotics prescribing to inform interventions about processes that can promote appropriate prescribing practice. We based this study on the perspective of the Dual Process Theory [11].

## Methods

### Design

Semi-structured open-ended telephone interviews with physicians and a one-time socio-demographic survey were used to provide insights into determinants of practice regarding a rational use of antibiotics in acute non-complicated self-limiting infections (common cold, bronchitis, sinusitis, tonsilitis, otitis media, cystitis) and propose explanations concerning identified influences and mechanisms of action [10].

### Context

ARena is an ongoing three-armed cluster randomized trial. It applies modern implementation strategies to enhance the appropriate use of antibiotics [10]. Across two German federal states (Bavaria and North-Rhine Westphalia), Arena is embedded into 14 primary care networks. These networks represent regional coalitions of physicians and other healthcare providers aiming for coordinated care of above-average quality [15]. ARena follows a complex implementation strategy with multiple interacting intervention components to address physician, ambulatory care team and patient knowledge and attitudes about the use of antibiotics [10]. Each arm received a different set of intervention components comprised of e-learning on communication, quality circles and data-based feedback for physicians and non-physician health professionals – comparable to medical assistants in USA [16] - information campaigns for the public, performance-based additional reimbursement, a computerized decision support system and culture-sensitive information material for patients in print and digital format on tablet computers to be used in waiting areas. An added cohort based on claims-data reflects standard care. The trial is accompanied by a process evaluation [10].

### Sampling and recruitment

No formal sample size calculation was done for the interview study as data were collected until saturation was reached. Using a purposive sampling strategy, 27 physicians were recruited through the ARena study team at the Department of General Practice and Health Services Research, University Hospital Heidelberg between March and May, 2018. The strategy supported the identification of individuals who were especially experienced in the phenomenon of interest and enabled detailed exploration and understanding of central themes and relations to specific experiences, roles and behaviors [17]. Eligible were all physicians participating in ARena. Written informed consent had to be signed prior to the interview.

Recruitment followed a structured procedure aiming for even distribution regarding gender and intervention groups. Out of 193 participating practices with 303 eligible physicians, a randomized sample of 40 physicians per intervention group were invited by e-mail via the aQua Institute, Goettingen, to participate in an interview (*n* = 120). A reminder was e-mailed after three weeks. Due to a disproportionately high number of participants from Bavaria, 11 non-responder physicians located in North-Rhine Westphalia received a second reminder after 12 weeks. All invitees received a personalized cover letter supplemented by written information detailing the study and the process evaluation. A feedback form had to be returned by fax or e-mail to indicate willingness to participate. All interested parties who met the inclusion criteria received a second personalized cover letter, the written information, a consent form and the socio-demographic survey form via postal service. All interested parties who sent a signed letter of intent to participate in an interview were contacted by phone to provide further information referring to the study and subsequently could be included in the process evaluation.

### Data collection

Data were collected by open-ended, semi-structured telephone interviews with physicians and a one-time socio-demographic survey. All interviews were conducted and digitally audio recorded in the first months (April to June 2018) after start of the ARena intervention. Based on a literature review and defined research questions, an interview guide was developed (see Additional file 2 for a translated version). The first two interviews served as a pilot; after this, minor adjustments were made where considered appropriate. All interviews were conducted by three researchers of the study team at the Department of General Practice and Health Services Research, University Hospital Heidelberg. During and after the interviews, additional notes were taken to document participant suggestions with regard to aspects of intervention delivery. All qualitative data generated were included for analysis. Participants received a small reimbursement fee after completing the interview and the socio-demographic survey.

### Data analysis

Pseudonymized verbatim transcripts were coded using a framework analysis [18] based on the Tailored Implementation for Chronic Disease (TICD) framework which classifies determinants of implementation in 7 domains: Guideline factors, Individual health professional factors, Patient factors, Professional interactions, Incentives and Resources, Capacity for organizational change and Social, political and legal factors [19]. The interprofessional team of three researchers (Public Health and Health Services Research) identified themes of interest deductively a priori from the TICD framework and the interview guide as well as inductively de novo from the data itself during the analysis. Independently, all researchers coded all transcripts iteratively. Intercoder congruity was facilitated by continuously discussing divergent codings, thus achieving the widest consensus possible, minimization of research bias and the risk of losing relevant content.

Trustworthiness of analysis and findings was ensured by following methodological strategies such as seeking for similarities and differences across and within accounts to ensure representation of different perspectives. Charting the participants’ views with regards to identified themes facilitated comparisons within and across interviews [20] and enhanced the transparency of the analysis. MAXQDA Analytics PRO 18 (Release 18.1.0) was used to organize and manage the data.

**Fig. 1:**
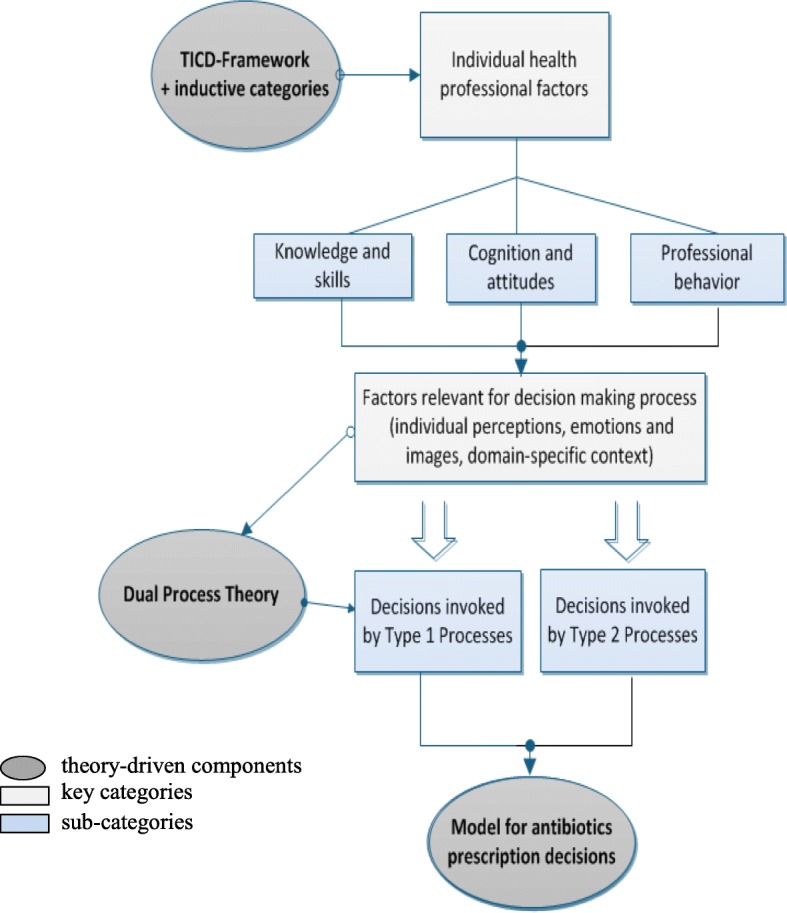
Flow of the analytical process

Socio-demographic characteristics (see Results Table 2) were analyzed descriptively using IBM SPSS Statistics Version 24 to describe the study population and facilitate assessment of generalizability. Subsequently, the approach of the Dual Process Theory [11–13, 21, 22] was applied to enable a profound understanding of the scope of individual health professional factors that induce dysrational prescribing decisions, or promote rational ones respectively. Fig. 1 illustrates the flow of the analytical process. Greyed ovals represent theory-driven components.

## Results

### Overview

Within the TICD domain of ‘Individual health professional factors’, results outlined below are structured with a focus on individual perceptions, emotions and images and domain-specific context. This reflects the identified scope of factors relevant to primary care physicians’ decision-making process regarding antibiotics prescribing for acute non-complicated self-limiting infections.

The code system matrix extracted from MAXQDA (Table 1) reflects the overall thematic framework for the analysis and indicates the proportional distribution of key themes by symbol size. Findings are presented with an indication of how frequent aspects were brought up. Extracted quotations are included for illustration. A supplementary table provides additional quotations [see Additional file 3]. All cited quotes were translated into English with due diligence and are referenced with participant number and transcript position.

**Table 1:**
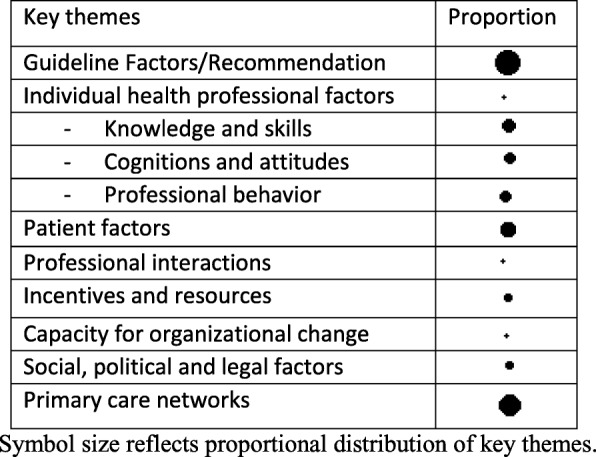
Code system matrix with main categories of the thematic analysis

Symbol size reflects proportional distribution of key themes.

A total of 27 primary care physicians (9 female, 18 male) participated in the interviews, ranging in age from 43 to 66 years. They were general practitioners (*n* = 16), internists (*n* = 6), ear-nose-throat specialists (ENT) (*n* = 3) and pediatricians (*n* = 2) with a mean of 26 years working experience. The sample represented the study population of physicians participating in the ARena study and reflected their overall demographics. All interviews were conducted as scheduled and no interview had to be aborted. When data saturation was reached, no further recruitment efforts were made. The response rate of 22.5% was to be expected in qualitative research involving physicians in primary care and considered satisfactory. Interview duration varied between 7:54 min and 62:50 min, with a mean duration of 28:14 min. Characteristics describing the participating practices provide indication of determinants of practice (Table 2).

**Table 2:** Characteristics of primary care practices participating in this qualitative study

Practices		Physicians (*n* = 27)
Practice type (n) (%)	Single practice	12 (44.4)
	Group practice	12 (44.4)
	Shared rooms^a^	2 (7.4)
	Medical center	1 (3.7)
Organizational changes during last 2 years (n) (%)	Affirmative	22 (81.4)
Number of patients per quarter of year	500–1000	6 (22.2)
	1001–1500	11 (40.7)
	> 1500	10 (37)
Estimated percentage of patients (%range) (n)	with migrant background	1–80 (27)
	receiving welfare benefits	0–50 (26)

^a^Separate financial entities and indemnity insurances, but shared rooms, equipment and staff

### Individual perceptions

Detailed accounts of ambulatory healthcare provision for acute non-complicated self-limiting infections provided insights into the complexity of a multitude of factors relevant to the process of taking therapy decisions. All physicians gave detailed accounts of their individual perceptions on how they habitually proceed when taking patient history for non-complicated infections. They mentioned which parameters they look to assess before taking a therapy decision and when uncertain. Among those considered important were the perceived condition of the patient, knowing the patient or not, lab parameters, family history and co-morbidities. Upon patient presentation and history taking, physicians’ pattern processing sought to detect factors to be associated with relevant prior experiences. Either it recognized a perception-driven and context-dependent need for a specific choice of therapy, or it did not. If recognized, according to the Dual Process Theory, fast automatic Type 1 processing was engaged. If not, slower, analytical less error-prone Type 2 processes were activated. In both cases a decision for or against an immediate or delayed prescription of antibiotics was taken.

Also, the importance of professional experience was mentioned. All physicians considered themselves to be very restrictive and reluctant with antibiotics prescriptions, even before taking part in the study.

“I take a look at the patient, do a physical exam and then, based on the taken history, I see whether it is bacterial or viral. A year ago, I started with drawing blood to assess CRP [c-reactive protein] and other hemogram parameters and avoid a potentially dangerous course. I do this generously when I am not certain.” (Physician #07, 0:51).

“I think we were very restrictive with prescribing antibiotics to begin with. Generally, it depends on the patient’s condition, the clinical presentation, simply how he feels, I think experience plays a major role, you just see: Ok, the patient sits across from me and he does not appear to be seriously ill.“(Physician #22, 0:44).

### Emotions and images

#### Perceptions prompting dysrational overrides

All interviewed physicians knew of and reflected on situations of deviating prescription choices. They named justifying reasons for their occurrence. Drawing on preconceived ideas, prior experiences, hear-say or ‘gut feeling’, physicians assumed patients of specific age groups, cultural or migrant background were expecting to receive a prescription for antibiotics when presenting with a non-complicated infection.

” We see a lot of patients from France, Spain or Russia or Hungary, they sometimes have a higher expectation of getting a prescription for antibiotics, at least that’s my impression. “(Physician #15, 02:04).

Physicians perceived a lack of health literacy in older patients. They also considered them to have concerns about their recovery if not given antibiotics. Younger patients were assumed to be willing to take antibiotics to be able to go to work. There was acknowledgement of prescribing antibiotics to persistent patients who would not back down: in some cases, because of feeling powerless, in other cases to avoid losing the patient to a different practice. These prescription choices against better judgement were considered to be exceptions.

“If he thinks he needs it desperately and I cannot convince him, to make sure he does not run to the next [doctor], I tell him: Here is your prescription for antibiotics that would be appropriate, but I don’t think you need it. Take it home and see how you feel during the next few days, wait for two, three days. But I have to say, this is a very rare situation among my patients. “(Physician #09, 05:56).

Physicians reported to resort to the strategy of delayed prescribing in situations of diagnostic uncertainty and limited potential for follow-up. They were certain about patients following their recommendations and assumed most would not fill a delayed prescription. Occasional patient feedback reassured them. All physicians who used delayed prescribing saw it as an exception to the rule. Six physicians considered the strategy inappropriate and did not use it at all, as they felt this would unduly shift responsibility for therapy decisions to patients.

” In rare cases when there are relapsing problems, when I know the patient and he is travelling, then I might use it, I might have used it once last year, really hardly ever. “(Physician #07, 11:44).

“So he has to come back two days later, then I take history again and take a new decision. I don‘t do things like that, either he is sick or he is not, we don’t prescribe antibiotics just for fun. “(Physician#19, 4:49).

Driven by insecurities, dysrational immediate or delayed prescriptions of antibiotics also occurred in situations of exceptional circumstances. Triggered by emotions and biased pattern recognition, such situations encompassed a limited potential for follow-up visits. This included upcoming weekends, public holidays, planned vacations and weekend on-call services. Efforts to reduce diagnostic uncertainties were reported - for instance by using point of care testing for CRP - as was the provision of antibiotics prescriptions before reaching a definitive diagnosis. Three physicians mentioned emotional concerns and a guilty conscience when not administering a treatment at all or recommending non–prescription medicinal products patients would have to pay for in full at the pharmacy.

“I might use it [delayed prescribing] when I am uncertain, when symptoms are borderline or circumstances are sensitive, e.g. the weekend, … I quite like it and patients very happily accept it. “(Physician #16, 02:46).

#### Perceptions prompting rational overrides

Thematically related quality circles enabled communicative in person peer exchange about the subject matter, experiences and approaches. They gave opportunity to refresh existing expert and guideline knowledge to participating physicians. Physicians applied communication strategies provided through the e-learning component in the ARena study. This boosted reflecting on habitual approaches. Addressing the subject of antibiotics prescriptions and corresponding expectations in the beginning of the consultation was found to establish clarity and produced unexpected patient reactions.

“Through the e-learning in ARena I learned that we as physicians think patients want antibiotics, but when I address it directly: So you think you would be served far better with antibiotics? Then they say: Oh no, I don’t want them at all. “(Physician #20, 03:17).

### Domain-specific context

#### Guidelines

Physicians were aware of guideline recommendations referring to non-complicated infections and antibiotics prescriptions. They considered guidelines to be important for their therapy decisions and strengthening to their own stance. At the same time, they shared critical views towards guidelines in general or about their transparency. Professional experience was noted as a guideline overriding factor.

“Guideline recommendations? In principal, yes, they do have an influence, but I have my own ideas there, I have always handled it the way I think is appropriate. “(Physician #19, 02:19).

#### Self-efficacy

Participating physicians reported several aspects of perceived self-efficacy for the context of decision-making regarding antibiotics prescribing choices. Among those were enabling a shared decision by discussing therapy options with patients, ability to persuade patients to agree to a proposed therapy option, patient trust into the experienced physician, and educating patients about the appropriate use of antibiotics. While three physicians stressed that they would decide about the therapy, not the patient, others also reflected on cases where a patient was considered not reachable by sound arguments and antibiotics were prescribed inadequately as a consequence.

“Usually I am lucky and I rarely have big difficulties, thanks to my persuasiveness and my medical trustworthiness when I say: No, first we will try without antibiotics. “(Physician #14, 03:29).

“I explain to them why, for what reason, why not, and 95% accept that and 5% go to see another doctor. “(Physician #02, 02:04).

“If he insists… if I cannot dissipate his concerns, I think he should get the antibiotics. “(Physician #23, 07:29).

#### Self-monitoring and feedback

Insights were given into approaches to self-monitoring. Physicians reported about engaging in auto-feedback, reflecting on previous experiences, and keeping their own statistics regarding antibiotics prescriptions. Also, they acknowledged that participating in the ARena study supported their efforts of analysis and reflection of their own decisions and enabled comparisons with other physicians’ prescribing choices.

” All I can say is that through participating in the study and the interview … I simply reflected myself again. This self-reflection just occurs and you broaden your perceptions and align them again, you question yourself. “(Physician #24, 17:06).

### Embedding results into the theoretical frame

**Fig. 2:**
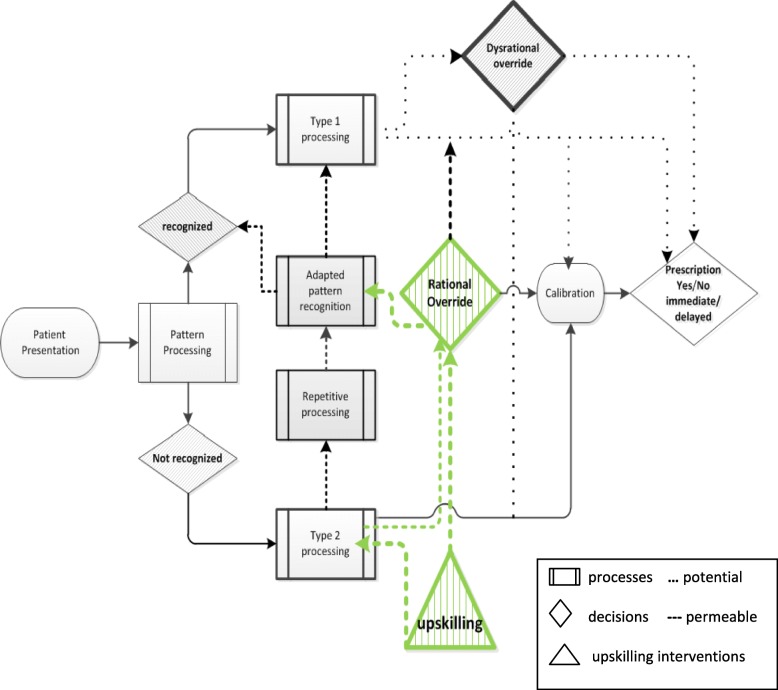
Model for antibiotics prescription decisions based on Dual Process Theory (adapted from [20, 22, 23]

According to the Dual Process Theory, based on recognized images, patterns and emotions, Type 1 processing can either result in an immediate decision, a fast but intently calibrated choice, or a ‘biased’, dysrational override of the physician’s expert reasoning. The latter can lead to prescribing decisions predominantly based on individual physician’s prior experiences and perceptions of patient characteristics such as age group, cultural background, expectations and intentions. Footing on the presented findings from the qualitative data, a theory-driven model is proposed to support their classification regarding physicians’ decision-making processes. The shown model for antibiotics prescription decisions based on the Dual Process Theory (Fig. 2) is adapted from a model for diagnostic reasoning [13, 14, 22] and runs from left to right and dynamically. It illustrates reciprocity, adaptability and permeability between the two types of processing. With regards to participating physicians’ antibiotics prescribing choices, this model indicates the crucial role not only of dysrational, but of rational overrides as well by illustrating upskilling intervention components can empower Type 2 overrides and adapt pattern recognition by providing new experiences.

Type 1 processing is fast, domain-specific, autonomous and ‚biased‘, with the potential of decisions being driven by perceptions and exceptional circumstances. Type 2 processing is slower, systematic and analytical and can be empowered by education, training, re-training and upskilling efforts. Both processing types can override the other. The model runs from left to right and dynamically, demonstrating reciprocity, permeability and adaptability.

In this study, Type 1 override choices of prescribing antibiotics lacked a consideration of indication, expanded the arbitrariness and variability of care, risking unequal treatment and consequently an increase of antimicrobial resistances. Type 2 processing cautiously and systematically considered all known and relevant aspects first before calibrating a decision.

While both processing types could override the other, rational overrides triggered by Type 2 processing lead to adapted pattern recognition and diffused as new domain-specific experiences into Type 1 processes. Type 2 overrides were empowered by intervention components targeting this potential by re-training, upskilling, re-sensitizing and creating new experiences and knowledge. Exemplary intervention components are e-learning on physician-patient communication approaches and quality circle attendance as offered and utilized in ARena, Point-of-Care testing or encouragement of follow-up visits.

## Discussion

This study analyzed qualitative data generated through semi-structured interviews with primary care physicians to shed light on factors crucial to antibiotics prescribing choices for non-complicated infections. Findings referring to inappropriate, non-indicated antibiotics prescribing against better knowledge were integrated into a theory-driven model to provide transparency as to how such dysrational prescribing decisions occur and to suggest remedy.

Previous research found that physicians’ perceptions of patients’ expectations may be a reason for inappropriate antibiotics prescribing [24]. However, clinical decision-making is a multifactorial and complex process in which many factors can play a role [25]. Research already has identified many factors associated with antibiotics prescribing in primary care [26–30]. To advance transparency of the complex phenomenon of dysrational antibiotics prescribing decisions, this study applied the concept of pattern recognition and the Dual Process Theory. The resulting heuristic model was adapted from a model for diagnostic reasoning by Croskerry [13, 22, 31] (see Additional file 1 [32]) and presented here embedded in its theoretical frame (see Results, Fig. 2).

One of the main concepts emerging comprised individual health professional factors relevant to the decision-making processes regarding antibiotics prescriptions for acute, non-complicated self-limiting infections. The identified scope of individual perceptions, images and emotions and domain-specific context became particularly apparent in reports about situations of limited potential for elimination of diagnostic and circumstantial uncertainties. At the initial point in consultations, physicians already sought to detect factors to be associated with prior experiences gained during their years of training and practice, routine and expert knowledge. This led to calibration of the perceived severity of patient condition and discomfort and rather simplistic heuristics about patient types and external circumstances before taking a therapy decision. Physicians faced uncertainties that made them susceptible to unconscious influences and personal biases. With regards to acute, non-complicated infections, upskilling evidence-based intervention components enabled more rational antibiotics prescribing decisions by supporting awareness and clear provider-patient communication.

All physicians saw themselves as careful, restrictive antibiotics prescribers who only occasionally made exceptions to the rules, usually driven by external circumstances and in some cases justified with concerns about administering expensive over the counter medication or no treatment at all. However, in this interview study, a noticeable dissonance was disclosed between physicians’ self-perception, expert knowledge and acknowledged dysrational antibiotics prescribing choices which were based on perceptions, intuitions and ‘gut feelings’ and lacked a consideration of indication. This supports the suggestion that aspects of emotion and compassion may constitute a bias for therapy decisions [33].

Dysrational immediate or delayed prescriptions of antibiotics were also evoked by exceptional circumstantial uncertainties such as upcoming weekends, public holidays, on call service or vacation periods thus expanding arbitrariness, variability of care and unequal treatment. Such choices were made as fast, unconscious Type 1 process decisions which are more likely to be inappropriate [34] and rarely get corrected [35], particularly as 86% of patients in Germany contact their primary physician only once for short term acute diseases [36].

While a simplistic relationship between expert knowledge, self-perception and actual prescribing choice is not to be assumed, antibiotics prescriptions against better judgement appear to be opaque particularly in situations governed by diagnostic uncertainties. Primary care physicians are routinely exposed to such diagnostic uncertainties. However, attempts to deal with uncertainties so far have been focused on how to reduce them, not on training physicians to manage or tolerate them [33]. Such uncertainties exert pressure on physicians [37], prompting them to rely and draw on emotion, intuition, prior experiences and simplistic heuristics without further validation. Resulting dysrational overrides in physicians’ expert reasoning - inappropriate prescribing - therefore constitute a challenge to care quality, potentially imperil continuity of care and lack in supporting the reduction of AMR drivers.

Although physicians need to use intuitive, automated cognitive processes in their daily practice, it is also important to know when and how to rationally adapt intuition and routines. A promising way to achieve this is by efforts of upskilling physicians [37] and their healthcare teams. The physicians’ critical view of guidelines can be considered as in line with findings from previous studies that indicate reservations about clinical guidelines among German General Practitioners [38] and physicians treating privately insured patients only [23]. This implies that guideline adherence does not come with ease. However, participating in a research project re-sensitized and strengthened physicians’ personal attitude towards prescribing choices.

### Strengths and limitations

The purposive sample of participants were especially knowledgeable about and experienced in the phenomenon of interest which enabled detailed exploration and understanding of a central theme and underlying perceptions. Structural variance was ensured through age, gender and years of working experience. Qualitative interviews are an important tool for researchers to facilitate and foster understanding of perspectives of targeted groups and telephone interviews are considered to be a valuable method of collecting information on sensitive topics [39]. All participants felt comfortable with this chosen method. It fit their busy schedules and they could remain in their own environment while being interviewed so no participant opted for a face-to-face interview. Rapport was built effortlessly and supported by vocalized acknowledgments deliberately given frequently during the interviews. This participant-centered approach allowed for consideration of verbal prompts and probes, follow-up questions and taking notes without influencing or distracting interviewees. Thus, applying this method resulted in rich data. Data analysis was guided by adequate methodological strategies to minimize research bias and to reduce the risk of losing relevant content. Typicality of observations was shown by providing simple counts where their support of the analysis can be expected and to meet potential notions of anecdotalism and exoticism. Reporting follows the recommendations of the COREQ checklist (COnsolidated criteria for REporting Qualitative research) [40].

However, some limitations for this research have to be reported. The method of open-ended semi-structured interview by telephone was chosen to provide room for open, candid answers, yet social desirability cannot be excluded completely. Interview duration might have been shorter than to be expected in face-to-face interviews. Visual cues of interviewees and their environment could not be included, but were not considered to potentially add valuable data to the study. Interviewees potentially held stronger opinions about the subject matter or were more aware of it than others not included in this research. Participating physicians were members in primary care networks already engaged in efforts of improving care quality and upskilling which possibly contributed to a selection bias. Variability in network abilities to foster complex processes requiring intensive professional education as found in previous work [41] was not investigated in this study. Aspects concerning cultural, societal or economic factors were not considered in this study either as the primary focus was on factors physicians potentially can change themselves. The mentioned aspects as well as the influence teaching roles might have on clinical decision making could be the subject of further research exploring such determining factors. Inherent in this type of research is a lack of external validity.

This paper describes that the interventions offered in the ARena study supported physicians in awareness about their own practice, introspection and in using new approaches in the provider-patient communication. Their Type 2 processing was successful in adapting pattern recognition and diffusing into domain-specific autonomous Type 1 processes by providing upskilling, re-sensitizing and new recognizable patterns. This demonstrates that although breaking habits might face difficulties, it can be achieved by targeting individual health professional factors with efforts of upskilling and creating new experiences. At the same time, it emphasizes the magnitude of continuous efforts of physician training and re-training with regard to rational prescribing choices. The proposed model can advance transparency of the complex phenomenon of dysrational prescribing decisions and inform future strategies and interventions to promote rational prescribing decisions.

## Conclusions

Physicians might face uncertainty when diagnosing patients and be susceptible to personal biases and unconscious influences. In these situations, cognitive errors are likely and dysrational overrides of expert reasoning might lead to undesirable quality challenging variance in care. With regards to a rational use of antibiotics for acute non-complicated self-limiting infections, evidence-based effective intervention components may provide upskilling, and potentially promote crucial rational prescribing choices that close an existing knowledge to action gap.

## Supplementary information

**Additional file 1.** A Universal Model of Diagnostic Reasoning. Croskerry, P. Academic Medicine84(8):1022–1028, August 2009. https://doi.org/10.1097/ACM.0b013e3181ace703. Model for diagnostic reasoning based on pattern recognition and dual-process theory. The model is linear, running from left to right. The initial presentation of illness is either recognized or not by the observer. If it is recognized, the parallel, fast, automatic processes of System 1 engage; if it is not recognized, the slower, analytical processes of System 2 engage instead. Determinants of System 1 and 2 processes are shown in dotted-line boxes. Repetitive processing in System 2 leads to recognition and default to System 1 processing. Either system may override the other. Both system outputs pass into a calibrator in which interaction may or may not occur to produce the final diagnosis. Copyright© 2019 by the Association of American Medical Colleges. Model reference kindly permitted by Pat Croskerry on April, 3, 2019 [32].

**Additional file 2.** Interview guide (translated).

**Additional file 3: Table S1.** Additional translated quotes extracted from the qualitative data.

## Data Availability

The datasets supporting the conclusions of this article are included within the article and its Additional file 3. All data that support the findings of this study are available from the authors upon reasonable request.

## Abbreviations

AMR: Antimicrobial resistance
CRP: C-reactive protein
ENT: Ear, Nose, Throat specialist
G-BA: Federal Joint Committee
TICD: Tailored Implementation for Chronic Disease framework

## References

[1] World Health Organization. Global antimicrobial resistance surveillance system (GLASS) report: early implementation. 2016-2017:2017 https://www.who.int/glass/resources/publications/early-implementation-report/en/. .

[2] World Health Organization. Global action plan on antimicrobial resistance. World Health Organization. 2015. http://www.who.int/iris/handle/10665/193736. Accessed 23 Apr 2019. 10.7196/samj.9644 26242647

[3] European Commission (2017). A European one health action plan against antimicrobial resistance (AMR).

[4] Bundesministerium für Gesundheit. DART 2020 – Antibiotika-Resistenzen bekämpfen zum Wohl von Mensch und Tier. 2015. https://www.bundesgesundheitsministerium.de/fileadmin/Dateien/3_Downloads/D/DART_2020/BMG_DART_2020_Bericht_dt.pdf. Accessed 23 Apr 2019.

[5] European Centre for Disease Prevention and Control. Surveillance of antimicrobial consumption in Europe 2013–2014. 2018. https://ecdc.europa.eu/sites/portal/files/documents/Surveillance-antimicrobial-consumption-Europe-ESAC-Net-2013-14.pdf. .

[6] Schwabe Ulrich, Paffrath Dieter, Ludwig Wolf-Dieter, Klauber Jürgen (2018). Arzneiverordnungs-Report 2018.

[7] German College of General Practitioners and Family Physicians (DEGAM). Leitlinien der DEGAM. 2019. https://www.degam.de/degam-leitlinien-379.html. Accessed 23 Apr 2019.

[8] Bundesamt fuer Verbraucherschutz und Lebensmittelsicherheit, Paul-Ehrlich-Gesellschaft fuer Chemotherapie e.V. Germap 2015 - Bericht ueber den Antibiotikaverbrauch und die Verbreitung von Antibiotikaresistenzen in der Human- und Veterinärmedizin in Deutschland. Rheinbach: Antiinfectives Intelligence; 2016.

[9] European Center for Disease Prevention and Control. Antimicrobial resistance surveillance in Europe 2015. Annual Report of the European Antimicrobial Resistance Surveillance Network (EARS-Net) 2017. .

[10] Kamradt M, Kaufmann-Kolle P, Andres E, Brand T, Klingenberg A, Glassen K (2018). Sustainable reduction of antibiotic-induced antimicrobial resistance (ARena) in German ambulatory care: study protocol of a cluster randomised trial. Implement Sci.

[11] Kahneman D (2013). Thinking, fast and slow.

[12] Evans JSBT, Stanovich KE (2013). Dual-process theories of higher cognition: advancing the debate. Perspect Psychol Sci.

[13] Croskerry P (2009). A universal model of diagnostic reasoning. Acad Med.

[14] Croskerry P, Nimmo GR (2011). Better clinical decision making and reducing diagnostic error. J R Coll Physicians Edinb.

[15] Agentur deutscher Arztnetze. Ueber Netze. Was sind Arztnetze? 2014. http://deutsche-aerztenetze.de/ueber_netze/was_sind_arztnetze.php. Accessed 14 Mar 2019.

[16] Freund T, Everett C, Griffiths P, Hudon C, Naccarella L, Laurant M (2015). Skill mix, roles and remuneration in the primary care workforce: who are the healthcare professionals in the primary care teams across the world?. Int J Nurs Stud.

[17] Ritchie J, editor. Qualitative research practice: A guide for social science students and researchers. 1st ed. London u.a.: Sage; 2003.

[18] Gale NK, Heath G, Cameron E, Rashid S, Redwood S (2013). Using the framework method for the analysis of qualitative data in multi-disciplinary health research. BMC Med Res Methodol.

[19] Flottorp SA, Oxman AD, Krause J, Musila NR, Wensing M, Godycki-Cwirko M (2013). A checklist for identifying determinants of practice: a systematic review and synthesis of frameworks and taxonomies of factors that prevent or enable improvements in healthcare professional practice. Implement Sci.

[20] Green J, Thorogood N (2018). Qualitative methods for health research.

[21] Djulbegovic B, Hozo I, Beckstead J, Tsalatsanis A, Pauker SG (2012). Dual processing model of medical decision-making. BMC Med Inform Decis Mak.

[22] Croskerry P (2009). Clinical cognition and diagnostic error: applications of a dual process model of reasoning. Adv Health Sci Educ Theory Pract.

[23] Zweigner J, Meyer E, Gastmeier P, Schwab F (2018). Rate of antibiotic prescriptions in German outpatient care - are the guidelines followed or are they still exceeded?. GMS Hyg Infect Control.

[24] Sirota M, Round T, Samaranayaka S, Kostopoulou O (2017). Expectations for antibiotics increase their prescribing: causal evidence about localized impact. Health Psychol.

[25] Murshid MA, Mohaidin Z (2017). Models and theories of prescribing decisions: A review and suggested a new model. Pharm Pract (Granada).

[26] Tonkin-Crine S, Yardley L, Coenen S, Fernandez-Vandellos P, Krawczyk J, Touboul P (2011). GPs‘ views in five European countries of interventions to promote prudent antibiotic use. Br J Gen Pract.

[27] Teixeira Rodrigues A, Ferreira M, Roque F, Falcão A, Ramalheira E, Figueiras A, Herdeiro MT (2016). Physicians‘ attitudes and knowledge concerning antibiotic prescription and resistance: questionnaire development and reliability. BMC Infect Dis.

[28] van der Velden AW, Pijpers EJ, Kuyvenhoven MM, Tonkin-Crine SKG, Little P, Verheij TJM (2012). Effectiveness of physician-targeted interventions to improve antibiotic use for respiratory tract infections. Br J Gen Pract.

[29] van Esch TEM, Brabers AEM, Hek K, van Dijk L, Verheij RA, de Jong JD (2018). Does shared decision-making reduce antibiotic prescribing in primary care?. J Antimicrob Chemother.

[30] Tonkin-Crine S, Yardley L, Coenen S, Fernandez-Vandellos P, Krawczyk J, Touboul P (2013). Strategies to promote prudent antibiotic use: exploring the views of professionals who develop and implement guidelines and interventions. Fam Pract.

[31] Croskerry P (2018). Adaptive expertise in medical decision making. Med Teach.

[32] Croskerry P. A Universal Model of Diagnostic Reasoning. 21.03.2019. https://journals.lww.com/academicmedicine/fulltext/2009/08000/A_Universal_Model_of_Diagnostic_Reasoning.14.aspx. . 10.1097/ACM.0b013e3181ace703 19638766

[33] Alam R, Cheraghi-Sohi S, Panagioti M, Esmail A, Campbell S, Panagopoulou E (2017). Managing diagnostic uncertainty in primary care: a systematic critical review. BMC Fam Pract.

[34] Croskerry P, Singhal G, Mamede S (2013). Cognitive debiasing 1: Origins of bias and theory of debiasing. BMJ Qual Saf.

[35] JBT SE, Frankish K (2009). In two minds: dual processes and beyond.

[36] Kraus EM, Pelzl S, Szecsenyi J, Laux G (2017). Antibiotic prescribing for acute lower respiratory tract infections (LRTI) - guideline adherence in the German primary care setting: an analysis of routine data. PLoS One.

[37] Lum EPM, Page K, Whitty JA, Doust J, Graves N (2018). Antibiotic prescribing in primary healthcare: dominant factors and trade-offs in decision-making. Infection, Disease & Health.

[38] Schneider Sandra, Salm Florian, Vincze Szilvia, Moeser Anne, Petruschke Inga, Schmücker Katja, Ludwig Norman, Hanke Regina, Schröder Christin, Gropmann Alexander, Behnke Michael, Lübke-Becker Antina, Wieler Lothar H, Hagel Stefan, Pletz Mathias W, Gensichen Jochen, Gastmeier Petra, Abu Sin Muna, Antão Esther-Maria, Behnke Michael, Boklage Evgeniya, Eckmanns Tim, Forstner Christina, Gastmeier Petra, Gensichen Jochen, Gropmann Alexander, Hagel Stefan, Hanke Regina, Hanke Wolfgang, Klingeberg Anke, Klimmek Lukas, Kraft Ulrich, Lehmkuhl Markus, Ludwig Norman, Lübke-Becker Antina, Makarewicz Oliwia, Moeser Anne, Petruschke Inga, Pletz Mathias W, Salm Florian, Schmücker Katja, Schneider Sandra, Schröder Christin, Schwab Frank, Trebbe Joachim, Vincze Szilvia, Vollmar Horst Christian, Walter Jan, Weis Sebastian, Wetzker Wibke, Wieler Lothar H (2018). Perceptions and attitudes regarding antibiotic resistance in Germany: a cross-sectoral survey amongst physicians, veterinarians, farmers and the general public. Journal of Antimicrobial Chemotherapy.

[39] Cachia M, Millward L (2011). The telephone medium and semi-structured interviews: a complementary fit. Qual Res Organizations Manag.

[40] Tong A, Sainsbury P, Craig J (2007). Consolidated criteria for reporting qualitative research (COREQ): a 32-item checklist for interviews and focus groups. Int J Qual Health Care.

[41] Brown BB, Patel C, McInnes E, Mays N, Young J, Haines M (2016). The effectiveness of clinical networks in improving quality of care and patient outcomes: a systematic review of quantitative and qualitative studies. BMC Health Serv Res.

