# Supplementary material for: The complex phenomenon of dysrational antibiotics prescribing decisions in German primary healthcare: a qualitative interview study using dual process theory
Source: Antimicrob Resist Infect Control. 2020 Jan 6;9:6. doi: 10.1186/s13756-019-0664-6 (PMC6945776; doi:10.1186/s13756-019-0664-6)
Supplement: Supplementary file 1 — Additional file 1. A Universal Model of Diagnostic Reasoning. Croskerry, P. Academic Medicine84(8):1022–1028, August 2009. https://doi.org/10.1097/ACM.0b013e3181ace703. Model for diagnostic reasoning based on pattern recognition and dual-process theory. The model is linear, running from left to right. The initial presentation of illness is either recognized or not by the observer. If it is recognized, the parallel, fast, automatic processes of System 1 engage; if it is not recognized, the slower, analytical processes of System 2 engage instead. Determinants of System 1 and 2 processes are shown in dotted-line boxes. Repetitive processing in System 2 leads to recognition and default to System 1 processing. Either system may override the other. Both system outputs pass into a calibrator in which interaction may or may not occur to produce the final diagnosis. Copyright© 2019 by the Association of American Medical Colleges. Model reference kindly permitted by Pat Croskerry on April, 3, 2019 [32]. [file 13756_2019_664_MOESM1_ESM.docx]

**Additional File 1**

[A Universal Model of Diagnostic Reasoning](https://journals.lww.com/academicmedicine/Fulltext/2009/08000/A_Universal_Model_of_Diagnostic_Reasoning.14.aspx)

Croskerry, P. Academic Medicine84(8):1022-1028, August 2009.
https://doi: 10.1097/ACM.0b013e3181ace703


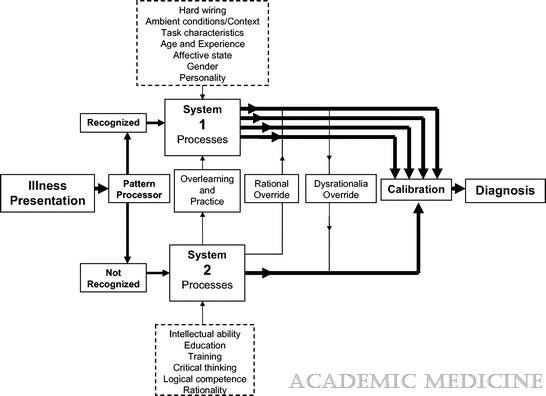


Model for diagnostic reasoning based on pattern recognition and dual-process theory. The model is linear, running from left to right. The initial presentation of illness is either recognized or not by the observer. If it is recognized, the parallel, fast, automatic processes of System 1 engage; if it is not recognized, the slower, analytical processes of System 2 engage instead. Determinants of System 1 and 2 processes are shown in dotted-line boxes. Repetitive processing in System 2 leads to recognition and default to System 1 processing. Either system may override the other. Both system outputs pass into a calibrator in which interaction may or may not occur to produce the final diagnosis. Copyright © 2019 by the Association of American Medical Colleges

Model reference kindly permitted by Pat Croskerry on April, 3, 2019.

[31]
