# Supplementary material for: The complex phenomenon of dysrational antibiotics prescribing decisions in German primary healthcare: a qualitative interview study using dual process theory
Source: Antimicrob Resist Infect Control. 2020 Jan 6;9:6. doi: 10.1186/s13756-019-0664-6 (PMC6945776; doi:10.1186/s13756-019-0664-6)
Supplement: Supplementary file 2 — Additional file 2. Interview guide (translated). [file 13756_2019_664_MOESM2_ESM.docx]

**Additional file 2: Interview guide** (translated)

**A – Use of antibiotics**

Describe regular course of action when taking therapy decisions for/against antibiotics regarding patients with acute non-complicated infections

- Influence of patient preferences
- Methods or strategies in use (delayed prescribing?)

**B – Effects of the quality improvement program with regard to patient care**

Support of therapy decisions by provided intervention components

- Which? / Why? / Reasons / necessary changes in daily practice
- Components not used / Tailoring of components to match workflow

Influence of study participation on therapy decisions

- Changes in attitude / Changes in physician-patient conversation

**C- Contextual factors – primary care network**

Structure and offers in your primary care network

Role of network for daily care practice / What has changed since becoming a member?

Reasons for becoming a member

How is peer exchange organized in your network?

- Frequency of peer meetings and exchange / support for daily practice (challenges)
- Information flow / Organisation of information flow
- Information exchange regarding use of antibiotics for acute non-complicated infections

Significance of network membership regarding own course of action concerning patients with acute non-complicated infections

- Possible explanation?

**D- Contextual factors – general**

Further significant factors /developments regarding care for patients with acute non-complicated infections and prescribing antibiotics

- Structural conditions / Organization of processes

**E- Closing**

Recommendations for future use of antibiotics in patients with acute non-complicated infections

- Ideas and remarks concerning a further development of the intervention program

What would you like to tell us besides already discussed topics?
