# Supplementary material for: The complex phenomenon of dysrational antibiotics prescribing decisions in German primary healthcare: a qualitative interview study using dual process theory
Source: Antimicrob Resist Infect Control. 2020 Jan 6;9:6. doi: 10.1186/s13756-019-0664-6 (PMC6945776; doi:10.1186/s13756-019-0664-6)
Supplement: Supplementary file 3 — Additional file 3: Table S1. Additional translated quotes extracted from the qualitative data. [file 13756_2019_664_MOESM3_ESM.docx]

**Additional File 3**

Supplementary Table 1: Additional translated quotes extracted from the qualitative data

| **Category** | **Key theme** | **Quote** |
| --- | --- | --- |
| Individual perceptions | Knowledge about own practice | “Taking patient history certainly is most important to me. … how long has it been going on, has it been present for a while or did it start recently, or is it a flare up. Depending on whether I know him or not, I ask about co-morbidities or recent antibiotics courses for a different illness. That is almost the most important thing, the rest is a bit of a confirmation. To me, taking history is the most important puzzle piece and then, how sick does the patient appear to be, and I ask him how strong his subjective perception of feeling sick is. Ears, nose, throat, lymph nodes, I always examine all of them, does not take much time, and I check the temperature. …. About 90% non-complicated infections, I tell the patient that clearly he does not need antibiotics.” (Physician #08, 02:35) |
| Emotions and images | Prompting dysrational overrides | “I discuss it less during on-call services over the weekend. If a patient comes in and tells me he has a purulent laryngitis, saw four ENT specialists and tried everything … then I don’t discuss that and say, OK, he should get antibiotics and then see the ENT again. These are rare moments. “(Physician #26, 03:36)  ”… sometimes, when the patient is insecure and the weekend is coming up, or he wants to take a vacation, then yes. But not in the majority of the cases.“ (Physician #18. 02:11)  ”Patients from France or Spain rather tend to them [antibiotics]. But I have to say that I don’t have any statistics. … My older son studies in Spain and there they like to get antibiotics …” (Physician#09, 06:50)  ”Reluctantly. I use this very rarely, because if at all, then I want to decide and this would shift the decision to the patients and that is difficult for them … .” (Physician # 06, 03:34)  ” Patients from Asia or southern Europe I would say expect a prescription for antibiotics to begin with. “(Physician #16, 04:40)  “I think generally with older patients, I am really rather more generous when it comes to antibiotics prescriptions. “ (Physician # 22, 04:53)  ‘Mostly young professionals who are under stress at work and think they get better." (Physician #03, 03:03)  ” Sometimes, if I get the impression that I hear something there, there is a bronchopneumonia or it could be one, or the patient feels immense pain, then I write a prescription for antibiotics. “(Physician #23, 00:34) |
|  | Prompting rational overrides | ”But basically, it was a little more eye-opening, how do I handle it?” (Physician #25, 8:32)  ” The quality circle is a real asset. The first one was really good, especially because you hear that others handle it completely different, how differing the experiences are, and questions came up immediately, very interesting. I took notes and they are on my desk now for my reference. “(Physician #23, 22:49) |
| Domain-specific context | Attitudes about guidelines | ”If there is a proven benefit for the patient, then guideline recommendations have to be followed strictly, let’s say for pneumonia, but if there isn’t, then: no antibiotics.“ (Physician #04, 16:52)  “Well, for urinary tract infections, there is a lot of confusion when you actually read the guidelines, because that leaves you clueless in terms of what to prescribe and this is certainly something that can be improved for the future. “(Physician #8, 34:42)  ”Guideline recommendations certainly empower my actions, because I can be certain: Ok, this patient does not need antibiotics. “(Physician #08, 04:32) |
|  | Self-efficacy | ”… our patients really reflect quite critically …one trains them [laughing]!” (Physician #06, 02:37)  ” If I have the feeling I don’t want to prescribe antibiotics, then I spread a little bit of the theory, so he won’t feel like being treated inadequately, but recognizes it is quite allright given the probability, and then there are no problems usually. In 95% of all cases, this meets acceptance.“ (Physician #16, 00:35) |
|  | Self-monitoring and feedback | “With some self-critisism you can always act more cautious, but you can’t always tell, because you don’t know if the patient went some place else.”  (Physician # 12, 29:15)  ” I try to treat in accordance with guidelines and to review my therapy decisions and strategies over and over again to assess for appropriateness. “(Physician #23, 5:03) |
